# “Vulnerable and strong at the same time”: Forcibly displaced young women’s lived experiences of their sexual and reproductive health and rights (SRHR) as unaccompanied girls seeking asylum in Sweden

**DOI:** 10.1371/journal.pgph.0005241

**Published:** 2025-10-09

**Authors:** My Opperdoes, Lisen Dellenborg, Gunilla Backman, Henry Ascher, Josephine T. V. Greenbrook

**Affiliations:** 1 School of Public Health and Community Medicine, Institute of Medicine, Sahlgrenska Academy, University of Gothenburg, Gothenburg, Sweden; 2 Knowledge Centre for Sexual Health, Västra Götaland Region, Gothenburg, Sweden; 3 School of Global Studies, University of Gothenburg, Gothenburg, Sweden; 4 Research Department, Angered Hospital, Gothenburg, Sweden; 5 Mason Institute for Medicine, Life Sciences and the Law, Edinburgh Law School, University of Edinburgh, Scotland; : St John's Medical College, INDIA

## Abstract

Unaccompanied girls undergoing forced migration are exposed to multiple risk factors affecting their sexual and reproductive health and rights (SRHR). Yet, little is known of their perceived SRHR needs. Thus, the aim of this study was to illuminate the lived experiences of SRHR needs of unaccompanied girls seeking asylum in Sweden, and capture how experienced needs are affected by the social contexts navigated pre, during, and post-migration. A qualitative constructivist grounded theory (CGT) methodology was applied. Data was generated through in-depth interviews with 16 participants aged 21–26, self-identifying as women, who experienced forced migration as unaccompanied girls. All had sought asylum in Sweden as minors (under age 18), and had been granted either temporary or permanent residence status. Data analysis was conducted in accordance with CGT. The findings show that unaccompanied girls and young women experience structural hindrances pre, during, and post-migration, including restrictive norms on gender and sexuality, various forms of violence, and exposure to racism and mistreatment in encounters with public institutions, impeding the fulfilment of their SRHR. Simultaneously, they construct pathways to protect themselves from harm, and find support in navigating and claiming their SRHR needs. To cope with daily struggles, and access SRHR, time, distance, and stability are important in recovery, and in adapting and integrating new perspectives on SRHR. In conclusion, this study shows that unaccompanied girls and young women are at significant risk of SRHR violations. The findings call for broad policy shifts, with focused protections against gender-based violence, in addressing the specific SRHR needs of this group. Moreover, evaluating and strengthening SRHR literacy among professionals within public institutions and these girls alike should be prioritised in promoting protective factors, independence, and agency.

## Introduction

Globally, displaced women and girls are vulnerable to having their sexual and reproductive health and rights (SRHR) violated. Fundamental to all, SRHR are defined as the right to the highest attainable standard of “physical, emotional, mental and social well-being in relation to all aspects of sexuality and reproduction, not merely the absence of disease, dysfunction or infirmity” [1]. Unaccompanied children, defined as individuals under age 18 separated from caregivers or legal guardians [2], are further made vulnerable [3]. Unaccompanied status often overlaps with being an *asylum-seeker* (seeking international protection in a foreign country), a *refugee* (having been granted international protection), and, in certain cases, being *undocumented* (living under threat of deportation, having entered and/or stayed in a country without legal status, or having been rejected asylum) [4]. Often, they are also labelled as *migrants* (a term encompassing all forms of migration) [2]. Unaccompanied girls are at increased risk of negative SRHR outcomes, requiring comprehensive support and protection when seeking refuge in an unfamiliar host country (the country where asylum is sought) [5].

UNICEF has reported that approximately 47 million children live in displacement, with 300 000 estimated to be unaccompanied (likely underreported) [6]. The number of unaccompanied children in Europe has been on the rise, and in 2023, 43 000 new cases were registered; approximately 10% were girls [7]. In Sweden, around 10 000 unaccompanied girls have sought asylum between 2000–2023 [8]. Yet, little is known of their SRHR needs. The aim of this study was to illuminate the lived experiences of SRHR needs of unaccompanied girls seeking asylum in Sweden, and capture how experienced needs are affected by the social contexts navigated pre, during, and post-migration.

### SRHR, adolescence, and displacement

SRHR are rooted in human rights, including the right to life, to health, to dignity, to education, to autonomy, to privacy, to family planning, to non-discrimination, to freedom from sexual and gender-based violence (GBV), and to access to effective remedy [9]. SRHR are protected through multiple treaties, codified in international, regional, and national laws, and in international political consensus documents [9–12]. States, therefore, carry a duty to promote and protect actions to change social norms, laws, and policies to uphold SRHR [1,9]. SRHR are also foundational to the Agenda 2030, Sustainable Development Goals 3 and 5, in ensuring health, promoting well-being, achieving gender equality, and empowering women and girls [13]. Crucially, universal access to SRHR requires integration into national strategies and programmes [13,14].

Promoting SRHR literacy is central in ensuring women and girls’ rights to freedom of choice in decisions that govern their bodily integrity, and access to adequate and adapted information and healthcare. As highlighted by Amanu et al. [15], sexual and reproductive health literacy is key in protecting and respecting SRHR, “especially among young people.” Sharma et al. [16] further emphasised SRHR literacy as essential in addressing inequities and mitigating power imbalances that impede the claiming and fulfilling of SRHR.

Social contexts also play a central role in realising SRHR, through sociocultural, institutional, and legal processes that shape individual experiences and relationships [17]. Norms about gender and sexuality shape in childhood, change over time, and vary between social contexts. Moreover, gender norms intersect with cultural perceptions on sex, ethnicity, migration status, social class, age, etc. [18]. Adolescence, defined as the developmental phase between ages 10–19 [19], commonly brings about ponderings on sexuality, identity, and sexual relationships. This phase is important in sexual development and exploration, and coincides with when expectations linked to gender norms intensify, and risks of harmful practises and experiences increase, particularly for girls [1].

Being displaced during adolescence compounds existing vulnerabilities [1]. Approximately one third of young people undergoing migration are estimated to have experienced sexual violence pre-migration [20,21]. Migrant youth with experiences of sexual violence also often refrain from reporting victimisation or seeking related healthcare [22]. Additionally, sexuality remains a topic surrounded by shame and taboo in many contexts, and speaking openly about SRHR, and accessing information, can be difficult [23].

Every child is protected under the Convention on the Rights of the Child (CRC) [24], and the United Nations (UN) has emphasised that specific and extended protections should be provided during displacement and unaccompanied migration [25]. Critically, risks of SRHR violations pre, during, and post-migration increase when children are unaccompanied [3]. Therefore, identifying factors that diminish these risks is essential in ensuring both SRHR and the CRC are upheld.

### Understanding the dynamics of SRHR during unaccompanied migration

Unaccompanied children are exposed to acute risks, and simultaneously, public institutions struggle in reaching them in preventative SRHR measures [26]. Repeated exposure to traumatic events [20], poorer sexual health, and difficulties in having their health rights fulfilled have also been reported [27]. Moreover, sexual risk is compounded by multiple factors, including low socio-economic status, social taboo, and school disruption [28]. This impacts SRHR literacy, and risks resulting in increased transmissions of HIV/STIs and unintended pregnancies [28–30]. Sexual violence further increases these risks, and deteriorates both sexual and mental health [31]. Additionally, in Europe, poorer overall mental health, including depression and post-traumatic stress disorder (PTSD), have been reported among unaccompanied girls compared to boys [32,33].

Research from the United Kingdom accents how transitioning to a host country involves multiple life changes, alongside adapting to a new society, language, and norms [34]. A Norwegian qualitative study found that even five years after arrival, unaccompanied children struggle to find belonging despite efforts to adapt, and often face prejudice and discrimination [35]. Balancing new relationships and preserving cultural identity also proves challenging [35].

Furthermore, transitioning from adolescence to young adulthood in the host country is characterised by sudden shifts, including often losing support systems upon turning 18 [35,36]. The Council of Europe [37] has emphasised that young refugees struggles in this transition must be acknowledged, as their successful inclusion in society is important in protecting them against existing vulnerabilities and GBV. How unaccompanied girls’ SRHR are affected by these transitions remains under explored.

### Structural vulnerabilities of being an unaccompanied girl

Primary reasons for fleeing unaccompanied include the death or persecution of a family member, war, trafficking, and violence [34]. Yet, some of the greatest threats to SRHR are structural, engrained in societal laws, norms, values, and institutions, and rooted in various forms of GBV [10,11,38]. GBV, and the term violence against women, are defined in Article 1 of the UN Declaration on the Elimination of Violence against Women as any act resulting in, or likely to result in, “physical, sexual or psychological harm or suffering to women, including threats of such acts, coercion or arbitrary deprivation of liberty, whether occurring in public or in private life” [39]. It also includes sexual harassment, or other non-contact forms of sexual violence, regardless of the relationship to the victim [38]. Critically, the UN Committee on the Elimination of Discrimination against Women (CEDAW) [11] has accented that GBV constitutes human rights violations, and thus states must be accountable in eradicating it.

GBV also includes harmful practices prescribed by contextually accepted norms, such as forced marriage, child marriage, and female genital mutilation [40]. Other forms of GBV consist of restrictions or denials of resources, impeding or restricting access to service and information, and crimes committed under the guise of ‘honour’, commonly referred to as honour-related violence (HRV) [40]. Women and girls are particularly at risk of GBV, such as physical, emotional, or sexual abuse, various forms of intimate partner violence (IPV), and domestic violence; all consequential to their dignity and health [28,40,41]. From an intersectional perspective, they are at risk of being made vulnerable and discriminated against by structural responses to their overlapping social positioning [42]. In the case of unaccompanied girls, this position is defined by them being a child, a girl, often a person of colour, and a migrant and refugee [42,43]. As stated by the UN Committee on Economic, Social and Cultural Rights (CESCR) [10], these overlapping positions may lead to being “disproportionately affected by intersectional discrimination in the context of sexual and reproductive health.”

Although research on unaccompanied girls’ SRHR needs is lacking, barriers to SRHR-related services experienced by migrant women are well documented; including limited awareness of pathways to access, financial barriers, distrust, fear, discrimination, and mistreatment [28,43–46]. Consequentially, migrant women are at increased risk of not seeking needed services [47–50]. Moreover, when understood through the lens of intersectionality, in being confined to marginalised positions in society, with limited access to protective resources, such as police, social services, and healthcare, migrant women are structurally discouraged from reporting or escaping GBV-related conditions [42]. Thus, fully capturing the lived experiences of SRHR needs of unaccompanied girls requires acknowledging GBV in its social context, as well as recognising the overlapping societal positions these girls are confined to.

### Knowledge gap on unaccompanied girls and their transition to adulthood

The lived experiences of unaccompanied girls and young women have been identified as an area in need of further research [51–53]. The present study builds on limited work exploring experienced conditions and, more specifically, addresses a research gap on the perceived SRHR needs of this population. Further knowledge is needed to contribute to a more comprehensive view of unaccompanied adolescent girls and young women’s possibilities for enjoyable, safe, and secure sexual experiences [1]. This is critical in ensuring experiences are free from oppression, discrimination, and violence, and based on informed decision-making [1].

Sweden has adopted a broad national strategy and plan for action aiming to fulfil good, equitable, and equal SRHR for all [53,54]. Swedish governmental agencies have also highlighted the importance of involving migrants, such as unaccompanied children, in investigating their SRHR needs; including in the design and implementation of preventative interventions [55,56]. Simultaneously, increasingly restrictive migration policies have been implemented across the European Union over the past decade [57]. In Sweden, among many other measures, this has manifested in standardising temporary (over permanent) resident permits, and stricter family reunification policies [58], significantly impacting the lives of unaccompanied children. Thus, exploring lived experiences during these legislative shifts is also important in understanding potential impacts on unaccompanied girls’ SRHR needs.

The overall aim of this study was to illuminate the lived experiences of SRHR needs of unaccompanied girls and explore how perceived needs are affected by the social contexts navigated in the pre, during, and post-migration phases of life, including in their transition to young adulthood in Sweden.

## Method and materials

The study was conducted in accordance with Constructivist Grounded Theory (CGT) methodology. CGT explicitly relies on the co-construction of knowledge, and the belief that meaning is shaped and understood through interaction with others [59]. Through its qualitative lens, the approach illuminates social structures and processes, and how these link to social actions and interactions; to learn, understand, and interpret research participants’ views, reaping new knowledge and insight [60,61].

### Sampling and participants

The complex recruitment process (conducted by MO and HA) was both broad and targeted. Initially, MO organised referral group meetings with various actors experienced in interacting with unaccompanied children and youth; representing youth clinics, maternal care, migrant health clinics, family centres, and non-governmental organisations (NGOs). Participants were also recruited through communities on social media, informal leaders, public schools, student health services, and legal guardians across Sweden.

Sixteen participants, self-identifying as women, took part in the study. All had sought asylum in Sweden (the host country), having migrated as unaccompanied girls (under age 18) from Somalia, Eritrea, Afghanistan, and Syria. At the time of interview, participants were aged 21–26, and resided in both urban and rural areas across Sweden.

A heterogeneity in ethnicity, sexual orientation, and language was represented, along with variations in levels of pre-migration school attendance (spanning 0–12 years), and duration of stay in the host country (spanning 3–12 years). Seven held permanent and nine temporary residence status. Some had also had their asylum claims rejected, and spent periods undocumented. All but two participants had finished upper secondary school in Sweden, and four held university degrees or were university students. Fourteen were employed. Relationship status varied among participants. Eight were mothers.

### Data generation

Data generation spanned from June 2022-January 2024. MO conducted twenty-one interviews in total, with five participants taking part in follow-up interviews. Interview duration spanned from 47-138 minutes (average length: 72 minutes; total generated: 1445 minutes). All interviews were audio recorded and pseudonymised, before verbatim transcription (with the exception of one, where detailed notes were taken instead, upon the participant’s request).

**Fig 1:**
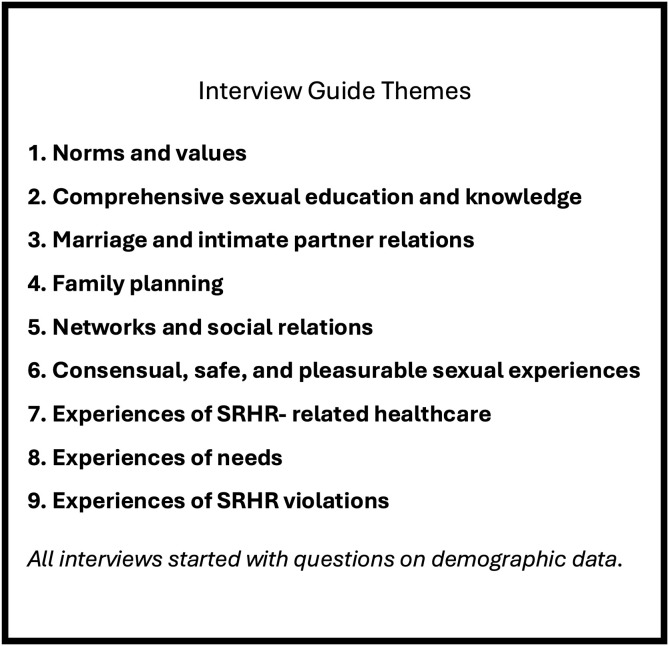
Initial themes explored in interviews.

CGT studies customarily employ unstructured interviewing, however, it was determined that developing a semi-structured interview guide minimised risks of posing questions that could be perceived as sensitive, difficult, excluding, or stigmatising. Applying the Guttmacher-Lancet Commission’s [1] definition of SRHR, and covering a broad variety of themes (see Fig 1), the guide was tested and refined. As a participatory component, referral group sessions were held with young adults who had experienced unaccompanied migration, professionals (including midwives, psychologists, cultural doulas, and counsellors), and NGO volunteers experienced in encountering unaccompanied girls. The guide was then continuously revisited and updated based on ongoing data generation and analysis, adding new questions to elaborate on unexplored areas, and test and validate potential theoretical findings.

Participants were free to choose interview format (eight chose in-person, three video call, and five via phone) and location. This was essential in respecting the need for confidentiality, while creating a safe and private space for sharing their experiences [62]. Consistent attention was given to signs of discomfort, facilitating pauses as needed. Participants were repeatedly reminded of their right to skip questions, or to retract or redact any answers given. To further promote safety, they were also offered to have a trusted person present during interviews (one participant chose her midwife).

All interviews were conducted in Swedish. Participants were offered an SRHR-trained interpreter, who was informed in advance on the interview’s aim and themes (three participants chose to have an interpreter present via phone).

### Data analysis

Data analysis began during data generation; starting with initial reflection and memo writing after each interview, and followed by coding data, asking analytic questions, and comparing and categorising data [61]. *Initial coding* [63] was conducted manually by MO and JG, where line by line was read and coded. The first two interviews were coded independently, before being compared to observe consensus and difference. Initial interviews were then discussed in the research team, to consider interdisciplinary perspectives. Further and continuous coding was completed in a tandem process, comparing notes between MO and JG. This provided closeness to the data and led to new analytic questions needing exploration in further interviews [61]. Moving forward, *focused coding* [63] was conducted using NVivo, and a sketch model for plotting and synthesising codes was developed by MO and JG to visualise shifting SRHR needs pre, during, and post-migration. Plotting and synthesising was subsequently conducted both individually, and in the team by MO, JG, LD and HA. Regular methodological meetings were also essential in formulating additional questions to capture nuance in narratives. *Theoretical coding* followed [61], where identified patterns were further interpreted and elaborated on by MO and JG. Central patterns were then tested through further data generation. This process included theoretical sampling [61], which also involved returning to available participants with new theoretically grounded questions, and requesting elaboration on earlier responses. This served as a means of testing and validating the findings [61].

Finally, a *storyline* was conducted by MO, to reach a higher level of abstraction [64]. This involved moving away from the data, abstracting the stories from how they were told in interviews, and broadening explanations of codes and categorised data. The storyline was subsequently refined and enriched by the team’s interdisciplinary perspectives. Then, a comprehensive empirically grounded theoretical model was conceptualised by MO and JG, abductively reconstructing all empirical categories through the lens of intersectionality. This final step was guided by constant comparison, moving back and forth between codes, categories, and theoretical concepts, while remaining guided by analytical and theoretical questions [61].

### Positionality and reflexivity statement

Our research team holds extensive experience engaging with questions of migration, sexual and reproductive health, paediatrics, socio-legal studies, and human rights - as researchers, lecturers, and clinicians. Therefore, positionalities held within the team have inevitably influenced methodological and analytical choices. Efforts to mitigate biases and strengthen validity were guiding in actively giving voice to the young women’s narratives. This involved continuously discussing data within the team, with a focus on how different positionalities offered different gazes and interpretations. Nonetheless, the team consists of European born, white (passing), academics, and thus, our lenses are inherently framed by these privileges.

Concerted efforts were made to apply an integrated interdisciplinary lens, requiring continuous individual and team reflection. *Reflexivity* was essential throughout this process in minimising disciplinary assumptions [61,65]. Further, practicing *methodological self-consciousness* was important; in reflecting on own beliefs and values, and maintaining awareness of one’s privileges, positions, and roles [66]. This also assisted in framing the researcher-participant relationship, and defining important intersecting factors, including power, identity, and marginality [66].

### Ethical considerations

Continuous ethical reflection took place throughout the research process. All parts were conducted in accordance with the Declaration of Helsinki [67], and ethical approval was obtained from the Swedish Ethical Review Authority (ref: 2021-06983-01).

All participants were adults (over age 18) at the time of recruitment. Before consent was sought, detailed information about the study was provided repeatedly to all participants. First, professionals or volunteers informed potential participants about the study. If interest was expressed, a meeting with MO was arranged where further adapted information was elaborated on to ensure informed consent. All written information about the study was translated into four commonly spoken languages among unaccompanied children seeking asylum in Sweden.

All participants were reminded of their freedom to withdraw at any time, before, during, or after interviews, prior to the seeking of informed consent in written or verbal form. Following all interviews, participants were provided MO’s contact information, in case unwanted emotions arose and support was needed. Contact information to a registered nurse external to the research team, experienced in encountering migrant women, was also provided. Moreover, if healthcare needs were identified before or during interviews, healthcare access was facilitated. This assistance was provided regardless of whether study participation ensued.

Finally, confidentiality was prioritised in communicating this research. While excerpts from all participants are represented in the findings, all samples of data are presented without being linked to specific participants.

## Results

See Fig 2.

**Fig 2:**
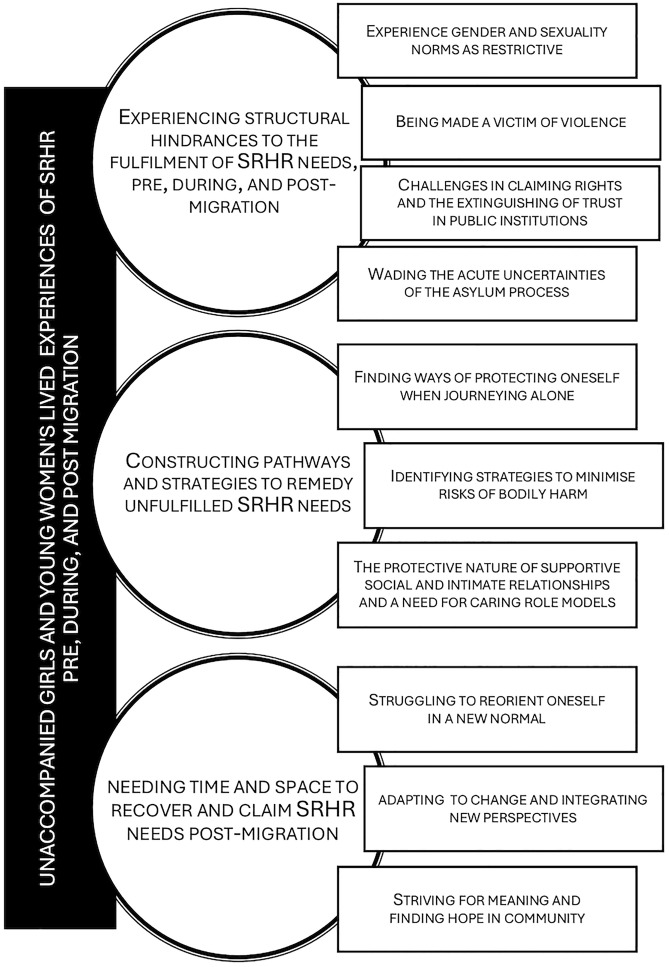
Categorical construction of the empirical findings. Empirically grounded categories illustrating the lived experiences of SRHR needs as unaccompanied girls and young women pre, during, and post-migration.

### Experiencing structural hindrances to the fulfilment of SRHR needs pre, during, and post-migration

#### Experiencing gender and sexuality norms as restrictive.

As young girls, many felt they were growing up out of place, in societies they perceived as strictly patriarchal. Many saw their opportunities limited because of their gender. Puberty intensified these constraints. At times, the girls broke norms, despite facing physical control or abuse. One young woman recalled her uncontrollable passion for sports: “I don’t control this, my heart does […] I hung out with boys, played soccer, and [my parents] would come get me and [simulates a slap] bang!” Normative expectations made the girls feel subordinate: “It was really really really hard to live like that, but that was my life.”

From an early age, the girls recalled being naturally curious about reproduction, sexuality, and relationships. While now being aware that this form of curiosity is entirely normal, at the time, exploring lust could lead to conflicting emotions: “I was deeply [religious]. When I felt I had to [masturbate], I did, but then regretted it. Thinking, ‘I’m a bad person, I must stop’ […] but I couldn’t control myself [laughs].” When exploring lust, being silenced or physically punished by their parents or others gave rise to further shame and confusion. Social norms could also strongly connect sexual relationships to marriage.

Upon fleeing, they were confronted by normlessness: “No one cares there [country of transit]. There are no rules.” Previously held norms no longer applied, and they became responsible for their own survival. To facilitate border crossings, their bodies could be objectified:

“At 16, a girl is made to dress in ways never previously allowed […] to draw attention, or neutralise it […] That clash […] those norms that I’d learned my entire life […] Suddenly, you’re made to wear this super short miniskirt.”

Arriving in the host country, they had to navigate foreign norms and values. They were met with racism, often reproducing restrictive gender norms and exotifying myths: “[The foster family] said, ‘you can’t ride a bike because you will break your hymen’.” She decided to call her mum to ask for advice “She explained, ‘it only [breaks] through intercourse or tampon use.” Later she found out that both were misinformed. Some gendered expectations in foster care were perceived as being more restrictive than those with which the girls were raised: “[Mum never expected] girls to behave girly […] we fought with boys, talked like boys […] Our mother actually just let us be children.”

Post-migration, discussing SRHR-related topics with others with similar backgrounds was also difficult, particularly when views on sexuality and sexual relationships deviated from those common in their countries of origin. Fearing rumours, some adapted what they shared and with whom.

### Being made a victim of violence

Most participants fled their countries of origin due to SRHR or GBV-related violations. One young woman shared what happened after she was raped as a girl: “[My parents] totally panicked. [A friend] said to leave fast […] ‘I’ll drive you, people are coming’ […] Commonly, they’ll stone the girl.” Several escaped forced marriages. Others received death-threats following being made victims of sexual violence, which was normalised, with victims often blamed. Additionally, reporting sexual violence to authorities entailed risks of further exposure: “[The police] wouldn’t help. Maybe they’d say, yes we can help but first [have sex] with me.” Some recalled being silenced when disclosing sexual assaults to their mothers: “Mum said […] ‘keep silent, don’t tell your father or anyone else.’” Others feared not being believed.

During migration, the girls’ experienced starvation, exploitation, and violence. Safety and support were absent. As one participant described: “I’d always been with family […] I wasn’t prepared to be alone, all alone, with strangers.” Trusting others was difficult, as even seemingly trustworthy people acted unpredictably. Everything felt like a surreal nightmare: “I landed in [transit country], and everything changed overnight.” They witnessed people die, younger children orphaned, drug use, prostitution, and people they loved being raped and murdered. Surviving the journey felt impossible and dependence on others became a matter of survival. Yet, dependence also increased risks of sexual exploitation, and objecting to exploitation could be dangerous. One participant shared how her employer became violent when she demanded her wages:

“One night, he unlocked our apartment and [raped] my friend […] It felt like chaos […] I couldn’t do anything because he [tied my hands and gagged me]. I couldn’t scream. She was bleeding heavily […] I didn’t know how to call the police, we didn’t have a phone. I didn’t know who could help us.”

Physical and sexual violence persisted post-migration in the host-country, including street harassment, groping, sexual assault, rape, and IPV. Disclosure, however, was rare: “[With my] psychologist, we talked about other stuff […] Sometimes you can’t tell anyone […] you’re ashamed.” A few filed reports, however, most cases were closed due to lack of evidence. One young woman who experienced HRV within her foster family said: “I was assaulted. I reported it to the police, but they did nothing […] My face was full of wounds, he nearly killed me, and threw me down the stairs […] They didn’t believe me.” Difficulties in meeting basic needs could also result in engaging in survival sex: “I was on the street. What could I do? […] He was very kind. I was grateful […] It wasn’t like he said I could live [at his place] without having sex with him.”

### Challenges in claiming rights and the extinguishing of trust in public institutions

Barriers to SRHR-related services and support were apparent throughout narratives. All participants believed that, in their countries of origin, adolescents had no access to SRHR-related services, and gynaecological care was only available to married women. During migration, access was also difficult. Some became pregnant, in some cases due to rape, but with little knowledge on pregnancy symptoms, healthcare was sought relatively late. One girl discovered she was pregnant in a transit camp: “‘It’s impossible!’ They said ’no, you’re [eleven weeks] pregnant […] You only have one week to decide if you don’t want it.’” Dependence on others for healthcare access and covering costs while on the move was common: “They said, if I wanted [an abortion], I had to go to the hospital […] The smuggler paid for everything.”

Access to comprehensive sex education pre, during, and post-migration was extremely limited. Some had received SRHR information upon arrival in the host country. However, this focused primarily on anatomy, STIs, contraceptives, and reproduction. Moreover, mixed-gender groups in the learning environment made it feel unsafe to voice questions. Having never discussed sex and relationships before, these topics were strongly associated with shame and taboo, leading several girls to avoid attending. In hindsight, they wished they had received information about their rights in intimate relationships. As one young woman shared, knowing her right to autonomy and consent in sexual relationships was the most vital thing she learned in sex education: “That no one can force me.” They also wished they had received more information about the host country’s norms and values, as well as contraceptives, LGBTQ+ matters, STIs, handling emotions, biological, psychological, and social perspectives on lust and pleasure, pregnancy, infant care, and motherhood.

Participants shared how limited SRHR literacy had exposed them to risks; unplanned pregnancies, STIs, and being trapped in destructive relationships and IPV. One girl refrained from disclosing she had begun menstruating, as she feared sharing with adults could lead to forced marriage: “If you have your period […] you have to meet a boy […] I feared I’d have to meet a man.” Limited SRHR literacy meant that proactive decisions were rare, and situations were simply managed as they arose; sometime detrimentally redirecting their life course:

“To know your rights, it really matters […] If I’d known that I could use contraceptives before […] I didn’t know they existed […] It wasn’t appropriate to have children in my circumstances […] I wasn’t in a good relationship, I didn’t have residence status, my life was so chaotic. So had I known, I could’ve used it. But no one told me there was such protection.”

Broadly, the young women expressed a want for supportive SRHR-related services, but had never been asked about their SRHR needs. A lack of trust in public institutions also represented a significant barrier to accessing necessary services. Limited rights literacy among professionals often exposed them to mistreatment, and fears of discrimination, racism, and dismissal led to distrust and avoidance in seeking help. This also led to feelings of unworthiness, hopelessness, and, at extremes, suicidal thoughts. Contrary to Swedish law, one participant suffering long-term complications due to genital cutting was denied essential SRHR-related care: “I can’t urinate, I can’t live like this […] I’ve been to every youth clinic in town […] They said,’if you’re undocumented, we can’t help you.’ […] It’s good to follow the rules, but when it’s someone’s life?”

Knowing their SRHR needs and being able to advocate for their rights when facing different authorities was seen as crucial. However, claiming one’s rights represented a constant and exhausting struggle: “You must continuously be assertive […] to feel the safety others feel. I have the same rights, but don’t feel that safety. It’s really important to know your rights, and know how to speak up.”

### Wading the acute uncertainties of the asylum process

During the asylum process, extended periods of uncertainty were experienced; due to rejected applications, temporary residence permits, or suddenly facing undocumentedness. Several girls struggled to survive daily. The asylum process itself deepened distrust, and sharing underlying reasons for seeking asylum, such as HRV, felt unsafe. Some girls were frequently relocated, preventing them from finding stability and establishing social bonds. Isolation further hindered them from seeking SRHR-related support. One participant shared: “I was silent the first five months […] I didn’t dare tell anyone [of severe menstrual pain]. I missed my mum.” Another said: “I don’t have anyone to talk to […]. First, I don’t know anyone, and second, I trust no one.”

Several experienced having their reproductive rights questioned due to their age and migration status, such as deciding if and when to have children. They felt deprived of rights others take for granted. One young woman shared how her right to have her partner’s support while giving birth was stripped from her when he was to be deported: “Doesn’t a child have a right to have their father there when they’re born […] Sometimes I think the rules are not for us [migrants] but for them [Swedes].”

Several of the young women had partners also in the asylum process. Some were detained in deportation centres, or had returned to countries of origin to apply for family reunification. Others, losing hope in Sweden, sought asylum elsewhere in Europe. Consequentially, families were split, significantly impacting the young women and their children.

### Constructing pathways and strategies to remedy unfulfilled SRHR needs

#### Finding ways of protecting oneself when journeying alone.

Displacement experiences varied among participants. Some were on the move for years, others for months or weeks. All had sought ways to protect themselves from sexual violence when journeying alone, and showed immense capacities to act in difficult situations. Finding support in others was essential; in communities of women, families, intimate relationships, or friends travelling alongside them. One participant described: “It’s so easy […] to get raped when you’re alone […]. So I always stayed with [other women].” Others avoided drawing attention to themselves. For some, having to rely on others whilst simultaneously striving for independence felt conflicting. Overcoming one day at a time took lead: “Just carry yourself [onward]. Just get on the next bus or train […] It’s really like having tunnel-vision.”

Some risked their lives to help others. One participant helped small children cross the Mediterranean Sea after witnessing their mother die along the journey: “[We] just tried to do what’s best for them, for us.” This, however, could come at great personal cost, leaving the girls stuck longer in transit countries, and exposing them to the violence of human trafficking.

Having survived the journey, many of the unaccompanied girls felt they could stand up for themselves and others: “I fear nothing.” Still, their courage could clashed with their outsider position in the new society: “[You feel] vulnerable and strong at the same time.”

### Identifying strategies to minimise risks of bodily harm

As girls, many had identified strategies to protect themselves from physical and sexual violence. Early on, they learned to avoid certain streets, and always ensured someone knew of their whereabouts. Before migrating, they were aware of the risks of sexual violence along the journey: “The only danger I was prepared for […] on the way to Europe […] was the risk of someone raping me.” Yet, this risk was not new:

“If I stayed in [country of origin], I’d never be seen as fully human and would be raped. If not today, maybe tomorrow, maybe next week. The risk is constant […] It’s better to be moving toward your goal [if you’re anyway going to] get raped.”

During migration, economic means could help secure safer accommodation. However, a constant awareness of risks remained:”Even [with] higher economic status, it’s very vulnerable being the only women at the hotel […] with all the men […] and no one knows [we’re there].”

Even post-migration, perceiving safety was difficult. Some found that new strategies were needed, as previously identified strategies no longer applied:

“If a boy ran after me, [my] strategy [was] to run between cars [across the street]. It’s really dangerous […] but you take the risk […] When I came to Sweden, I thought ‘oh no, the streets are really broad here […] He’d catch me in the middle of the road.’”

Several were placed in youth homes for unaccompanied children, sometimes as the only girl there. Feeling unsafe, they developed strategies to protect themselves from unwanted attention and sexual harassment, including dressing and behaving more boyish, isolating oneself, avoiding male staff, or entering an intimate relationship with a housemate.

### The protective nature of supportive social and intimate relationships and a need for caring role models

Throughout their lives, strong role models influenced their SRHR. Several participants describe their upbringing in their countries of origin as rather safe, surrounded by supportive parents, other meaningful adults, and friends. Some parents also publicly defended their daughters: “Every time I was hit [by teachers] at school, [my mother] went there and threatened [them].” Several girls witnessed adults questioning norms, and they were encouraged to marry for love, pursue education, and avoid financial dependence. Others felt parents feared for their safety due to their gender.

During migration, intimate relationships held great value and brought intense emotions. Some offered brief distraction, others longstanding solace and protection: “I was alone [and] it felt unsafe. What if I ended up somewhere […] But there, I met my husband.” A lust for experimentation relating to sexuality and intimacy was also expressed: “I was actually very curious, and wanted to do whatever I felt like. [I] wanted sexual freedom.”

Additionally, others offered key support along the way: “The interpreter gave me a business card, saying, ‘there’s an [abortion] doctor, he’s really nice […] He can help you.” In the host country, teachers, midwifes, foster parents, or legal guardians became meaningful role models: “She helped me a lot. She explained so much.”

Through conversations with friends and others, participants sought out support on sexual identity and orientation, sexual relationships, and handling unwanted advances. These discussions encouraged exploring one’s desires and body:

“My friend asked if I had masturbated […] ‘[If] you tell, I’ll tell!’ She said, ‘ok, I’ve done it a hundred times’ [laughs] and I said, ‘ok, I’ve done it too.’ My other friend said she hadn’t, but […] maybe she had, but felt ashamed […] I said, ‘try it, it feels good.’”

In the host country, conversations with others in marginalised positions, such as other girls of colour, and minority and activist communities (such as LGBTQ+ spaces), were also meaningful.

### Needing time and space to recover and claim SRHR needs post-migration

#### Struggling to reorient oneself in a new normal.

Migrating unaccompanied was traumatic, and marked by loss and severed ties. Post-migration, recovery was required. However, most described this period (still ongoing for some) as intense and exhausting, as they struggled to reorient themselves: “It’s an entirely new country, language, people […] I knew nothing. I was like a newborn baby when I arrived.” Adapting to the new social context demanded effort and engagement, and extreme fatigue replaced the high levels of stress experienced during their journey.

Some took pride in staying connected to identities that had shaped them, whilst also embracing the freedom to integrate new ways of being in the host country: “I like my culture [and] that I’m a Muslim woman. I want you to have that image of me […]. It says who I am […] an African woman, who’s a Muslim, and who does whatever she pleases [here] anyway.” For most, however, a strong desire for acceptance by the host society often came at the expense of their own identities.

Reorienting oneself in intimate relationships could also give rise to conflicting emotions; particularly surrounding trust and respect, and worries of being sexualised. Entering new relationships could feel overwhelming and depleting. Some also feared men, and doubted their own abilities to form romantic relationships: “Safety is a huge one […] I must be sure […] that nothing dangerous will happen […] Most thoughts about men scare me actually.”

As young women, exploring their own boundaries and understanding their partner’s was important. As adolescents, crossing or having boundaries violated led to regret and sadness. Setting boundaries was learned over time, and many now felt able to communicate their needs freely to their partner: “There is no fear or shame […] so you can say what you want.” This also allowed them to enter equal and respectful relationships, and leave harmful ones and IPV. One young woman described how SRHR brought autonomy: “For me, SRHR is a journey toward independence. It’s like my ultimate independence, when I have real full access to SRHR. Where I get to decide who I have sex with, simply put.”

Simultaneously, struggles in daily life impacted their ability to engage in intimacy: “During sex […] you remember something, problems you have, or uncertainties, and you lose [desire].” Voicing this, however, was not easy. With time, daring to show both strength and vulnerability assisted in communicating one’s own sexual needs more openly. It also allowed for safely exploring their partner’s needs: “To trust the other person, and feel it isn’t dangerous, comes [with time]. With good communication, talking, and getting along […] it’ll come.”

### Adapting to change and integrating new perspectives

Developing and integrating new SRHR-related perspectives required time. Landing, receiving support in handling trauma, and getting help with practical concerns were described as prerequisites to adapting. In most cases, this was delayed by asylum processes, as well as family reunification claims. While attempting to reunite with their partners (some, fathers to their children), family life felt suspended indefinitely. During these periods of existential uncertainty, their own SRHR needs were deprioritised and negatively affected.

As girls, some relied on their parents’ capacities in choosing a suitable partner. Post-migration this responsibility shifted: “I [still thought] parents decide who you [marry] […] Suddenly, I was responsible for deciding for myself.” Navigating new perspectives was a process, and with time, these were integrated. For example, many shared having shifted in their views regarding the freedom of all to choose their own identity, sexual orientation, and partner.

Distancing themselves from traumatic experiences required time and space. Trauma treatment and increased SRHR literacy facilitated this. Reading also helped in recognising feelings and interpreting experiences. One participant explained: “I read […] also to understand my own emotions […] I’d recognise myself in some article, or on YouTube […] It was so amazing to recognise myself in others.” Moreover, this also cultivated an awareness of contexts beyond their own. Some participants shared how being introduced to philosophical literature by their parents had also expanded their horizons: “I think it really came from the feminist literature and opinions, and a constant desire for justice and equality.”

Being given space, and the language to articulate what they had been through, they were able to reframe their traumatic experiences. As one young woman described, she could now shift blame away from herself for sexual abuse she had suffered: “If it happened again, I’d know it wasn’t my fault. I didn’t do [or choose] anything.” Moreover, when professionals asked about, and truly listened to what had happened to them, it eased the daily burdens of their past.

### Striving for meaning and finding hope in community

Although struggle dominated all narratives, feeling welcomed provided moments of respite and recovery: “It gave me a lot of hope.” Some girls had arrived before more restrictive migration laws were implemented, affording them swifter pathways to asylum and permanent residence permits. This enabled them to pursue education and family reunification without extended uncertainty:

“They treated me very well. Not like they are treating others now […] I came in summer […] and got [asylum and residence permit] really fast […] in time to start school right away, as I had always wanted […] I said I needed my family […] and got started with everything for [reunification].”

Moreover, opportunities to explore their own SRHR views and needs, and shape their identities, were found in communities that welcomed their differences and allowed them to freely express themselves; cultivating a sense of meaning, joy, and belonging.

However, for many, loneliness, isolation, and uncertainties about asylum outcomes and residency permits suspended recovery. One participant described being housed alone in a hotel, in early pregnancy, without social network, while her partner sought asylum abroad. Imagining a future, then, seemed impossible. For others, living in the moment was the only means of coping with the past. Socialising was difficult, particularly when energy levels were low, and joy felt out of reach. Sharing their past also required a deep sense of trust, and was inhibited by desires to protect others from painful stories: “My friends […] they easily get upset over small stuff […] If I told them [about what I have been through], they’d feel very upset.”

No matter the life stage, all highly valued independence and a stable income. While some had been given opportunities to accomplish these, others dreamed of stability, safe accommodation, employment, and access to education. All aspired to further their education, and most had already finished an upper secondary degree. A few were students in universities, or had finished their degrees and joined the workforce. Moreover, with the passing of time, an increased level of agency was experienced, contributing to a belief that a desirable life could be created. Reflecting on what had been overcome could also give strength: “I feel like, wow, I’ve been through all this.”

Several described their experiences as offering them a unique perspective and desire to help others affected by war, conflict, and displacement. One young woman’s engagement sparked when she served as an interpreter for other unaccompanied children: “Back then […] they brought in [SRHR] but we didn’t listen […] But [later] I learned while interpreting for them. I think it’s extremely important information.” A commitment to social justice, human rights, and equal treatment was also present. One young woman aspired to empower girls to live free from oppression and sexual violence:

“I want them to feel that they’ll make it in life, without a man […] And even if problems arise, sexually, or with a man, or girl stuff […] the solution is in her daring to leave […] and that there is always a way out.”

Contributing to the well-being of others was also perceived as meaningful and identity-shaping: “Maybe the main reason I’m in medical school is to use this knowledge and education […] to lift a burden from another human being, and see the impact.”

## Discussion

Our findings illuminate how, across social contexts, unaccompanied girls and young women’s SRHR are transcendently affected by vulnerabilities rooted in legal and social structures, norms, and values. These structural forces span from childhood to adolescence, through the migratory process as unaccompanied girls, and into young adulthood in the host country. While the findings are broad, this discussion will address our three main contributions: *Hindrances to SRHR at a structural level*; *violations of SRHR at an institutional level*; and *promoters and hindrances to the fulfilment of SRHR needs at an individual level*. Further, anchored in these empirical findings, and reconstructed through the lens of intersectionality, we introduce a novel theoretical model illustrating structural, institutional, and individual level processes, as well as how these intertwine to either support or impede the realisation of unaccompanied girls’ SRHR.

As our model demonstrates, structural forces significantly influence whether SRHR needs are met. Through the lens of intersectionality, this can be understood as a distribution of oppressors that construct hindrances to SRHR at *the structural macro level*. Unaccompanied girls face multiple oppressions that disregard that they are victims of forced migration. These emerge by nature of their overlapping social positions; being unaccompanied, a girl, a child, a person of colour, holding migrant status, financial insecurity, and limited access to education. Some face further oppressions due to sexual orientation, or suffering from short and long-term mental or physical difficulties. In all cases, structural oppressions demonstrate themselves most acutely as SRHR violations at *the institutional meso level*, in encounters within public institutions. This manifests as restricted access to services and support, and interactions with professionals being dominated by misinformation, exotification, racism, dismissal, mistreatment, and migration-related traumas. In turn, this forces unaccompanied girls and young women into positions of dependence, exposing them to conditions that increase the risks of GBV, further impacting their rights to dignity, safety, and health. At *the individual micro level*, to varying degrees, unaccompanied girls show agency and capacities for developing resilience and resisting oppression. They strive for independence, educate themselves, find or construct alternate pathways to access otherwise restricted support and services, develop SRHR literacy, claim their rights, stand up for themselves and others against injustice, and maintain hope for the future. Although they may hold high levels of agency, they can also simultaneously suffer from various migration and SRHR-related traumas hindering their recovery. Factors such as stability, access to trauma treatment, and support in reorienting oneself in the host society will also mediate capacities for resistance.

Notwithstanding their agentic counters to structural oppression, social support holds increased importance in having SRHR needs met. More critically, social support at a micro level can be a lifeline when opportunities are suffocated at institutional and structural levels. It can also be vital in leaving harmful relationships, IPV, and HRV. Moreover, tangible strides toward stability, security, and independence are essential. As shown in the findings, safe pathways to permanent residence status, family reunification, access to education and employment, and safe accommodation are paramount in recovering from both migration and SRHR-related traumas. Furthermore, these pathways promote resilience required in accessing and claiming SRHR. In contrast, being starved of such opportunities impede the fulfilment of SRHR, and generate social conditions where various forms of GBV will increasingly dominate. Moreover, promoters and hindrances to recovery and their effects on supporting or impeding the fulfilment of SRHR are not linear, as opportunities will vary between cases and over time.

**Fig 3:**
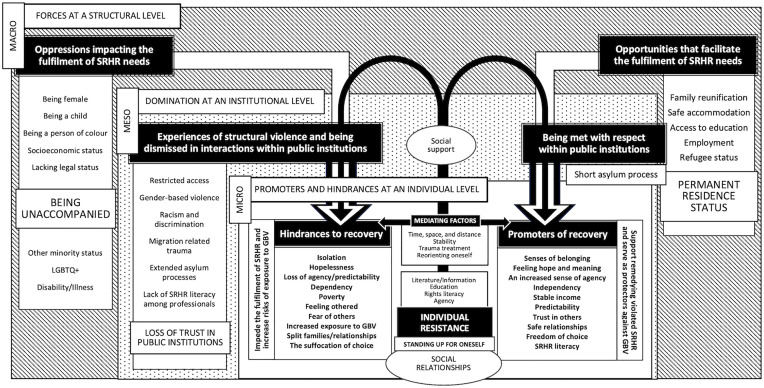
Theoretical model illustrating oppressions and opportunities affecting the fulfilment of unaccompanied girls’ SRHR needs. The figure shows how both oppressions and opportunities exist at a structural macro level, demonstrate themselves most acutely at an institutional *meso* level, and function as hindrances or promoters at an individual *micro* level. Oppressors to SRHR fulfilment dominate experiences of structural violence with increased risk of, for example, restricted access to public institutions, racism, mistreatment, and GBV, resulting in the extinguishing of trust. This in turn, hinders recovery and impedes unaccompanied girls from having their SRHR needs met. When structural opportunities exist, such as permanent residence status, family reunification, access to education, and economic stability, these facilitate the fulfilment of SRHR, allowing needs to be met and promoting recovery. Critically, such opportunities also support remedying SRHR violations, and protect from exposure to GBV. Yet, oppressions and opportunities are not linear, and vary pre-migration, throughout unaccompanied migration, with time spent in the host country, and with distance from traumatic experiences. Importantly, with existing structural forces, both individual resistance and social support in resistance become essential in claiming SRHR. Although unaccompanied girls can hold high levels of agency in resistance, they can simultaneously suffer from various migration and SRHR-related factors hindering their recovery. Additionally, factors such as stability, access to trauma treatment, and support in reorienting oneself in the host society further mediate capacities for resistance.

The main findings on unaccompanied girls’ and young women’s experiences of SRHR, violations of SRHR, and the factors that promote or impede their fulfilment are situated in a Swedish context. Yet many of these limitations arise already in countries of origin, and during the migration process. Although such experiences may vary with country of origin or migration route, their structural character makes them likely transferable across contexts. From an intersectional perspective, barriers to SRHR persist regardless of context, as they are rooted in overlapping forms of structural violence and oppression, including majority population norms and expectations. While it could be expected that opportunities for SRHR-related support would differ between welfare and non-welfare states (where access to family reunification, safe accommodation, education, employment, or refugee status has historically be inaccessible), the findings of this study show that even a welfare state such as Sweden has become increasingly restrictive. Where policy frameworks vary, the mechanisms by which structural barriers constrain SRHR access remain comparable, suggesting that the findings are likely transferable to other host countries in the Global North. The results may also be relevant to other populations, including girls and young women with differing experiences of forced migration, as well as those living in undocumentedness or with other precarious legal statuses (Fig 3).

Considering the many health and social factors integrated in SRHR, the impact of norms on gender and sexuality is particularly salient for young people [1]. As the findings illustrate, battling norms and values on gender, sexuality, and reproduction is an ongoing process, pre, during, and post-migration. Previous research has shown that girls, to a greater extent than boys, challenge and resist gender norms, due to inequalities enshrined within the norms themselves [68]. Unaccompanied girls, as shown throughout this study, are aware of, and struggle with unequal and unequitable conditions structurally assigned to their gender.

As the theory of intersectionality informs us [42], the overlapping of several marginalised social positions (as shown in Fig 3) compounds oppressions, and perpetuates domination and restrictions. In the case of our findings, difficulties in accessing public institutions, GBV, and having no safe or legal passage, confine unaccompanied girls to positions that *make them* vulnerable. To ensure their access to SRHR, underlying causes of oppressions and violations must be addressed. As accented by CEDAW and CESCR [10,11], our findings highlight that states must do more to implement and promote SRHR-informed laws, policies, and programmes, and actively work toward eliminating GBV for this group. Critically, public institutions must be equipped to ensure access to comprehensive and adapted SRHR-related services. Developing SRHR-informed support in recovering from GBV is also essential. Additionally, efforts should be made to raise awareness and to ensure safe migration pathways for populations at risk of SRHR violations. In line with previous research [69], our findings accent how being pregnant and on the move compounds challenges in accessing needed knowledge, information, support, and services. Improving access to SRHR during migration therefore also remains crucial.

As the findings show, SRHR literacy among girls and young women is fundamental in constructing pathways to realising their SRHR. However, while feeling empowered in claiming one’s rights can be a source of resilience, being burdened with the duty of having to do so represents a real struggle. As illustrated throughout the findings, these girls face racism and discrimination in encounters with authorities and professionals in social services, healthcare, childcare, and the asylum process. Lifting this responsibility from the individual level requires investing “into the structures, services, and policy environment” [16]. Ultimately, meeting unaccompanied girls’ SRHR needs remains the responsibility of the host country, and those encountering them in public institutions. Therefore, strengthening SRHR literacy among authorities and professionals is essential, and particularly critical for those in positions to assist unaccompanied girls and young women struggling in acutely vulnerable circumstances.

As highlighted by Sharma et al. [16], strengthening SRHR literacy requires taking into consideration how new resources reach women who need them, how these connect with their lives, and whether support needed to use and benefit from SRHR-related information is available. Further, SRHR literacy efforts must “reflect the ways in which women learn, where they learn best, and who they trust” [16]. Across contexts, SRHR literacy appears as largely absent, or poorly adjusted to the needs of unaccompanied girls. More starkly, we have presented several instances where a lack of literacy consequentially impacted life-altering decision-making, leading to increased risks of STIs, unplanned pregnancies, and difficulties in leaving unsafe relationships. This amplifies failures in educational efforts, where SRHR literacy appears to be addressed only superficially, if at all; leaving the individual responsible for sourcing information wherever possible. It also runs counter to the Swedish Public Health Agency’s expressed urgency in strengthening these girls and young women’s right to SRHR-related information in prevention work [48]. Thus, our findings highlight a need for both education and public health systems to take responsibility for ensuring that all girls and young women are offered comprehensive sex education tailored to their needs. It is paramount that unaccompanied girls be given special consideration, including being offered to attend without the presence of the opposite sex.

Previous calls for strengthening SRHR literacy stress the importance of also reaching out to meaningful adults and other community members who support migrant youth in decision-making [15,16]. When parents become unable to provide the support needed, other adults can step forward in their place [70]. As shown in our findings, being assisted by a safe adult, who takes on the responsibilities of a parent, can make a difference in whether unaccompanied girls’ SRHR needs are met. However, it is vital that adults placed in these positions of trust hold sufficient levels of SRHR literacy themselves, are informed on existing vulnerabilities, and are equipped to support the girls in claiming their SRHR where structural hindrances dominate.

It has been argued that stricter migration policies intensifies structural violence, leaving women at increased risk [71,72]. Further, factors such as migration status, age, and gender fuel inequalities that increase adolescent girls’ vulnerabilities [70]. Our findings illustrate acute examples of the lived impact of restrictive migration legislation implemented in Sweden in 2016 [58]. Consequentially, many of the young women who participated in this study were left with limited options, and little to no support from public institutions. This was particularly evident in their transition from adolescence to young adulthood, where being abandoned by the system upon turning 18 aggravated vulnerabilities and left them at risk of SRHR violations. More acutely, an increased dependence on informal pathways to accessing support, and a decrease in government assistance, compounded already existing vulnerabilities to GBV. Furthermore, as reflected in previous literature [36,73], our findings show how lacking legal residence status and material support amplify unaccompanied girls’ dependence on others. In turn, this contributes to isolation, and restricts access to public institutions, placing them at increased risk of exploitation.

Our findings also amplify the significant burden of the asylum process on unaccompanied girls and young women’s SRHR. Post-migration, predictability is crucial in navigating the new society [5,34,74]. Enduring and overcoming trauma, migration-related stress, and PTSD is immensely challenging when living with uncertainty surrounding the past, present, and future, due to rejected asylum applications or temporary residence permits [32,37,51]. Importantly, participants in this study who arrived in Europe before 2015 reported more opportunities for stability and personal development. Thus, the findings suggest that permanent residence, reunification with family, and access to education and employment all support recovery and independence. Conversely, we found that uncertainties surrounding asylum and refuge increased risks relating to SRHR violations, including infringements on the right to bodily autonomy, to choose partner, to safe and healthy family life, to sexual and identity development, to information and education, and to health [9]. Many of these violations also breach rights enshrined in the CRC [24]. In line with a vast amount of previous research in adjacent areas, our findings accent the need for decision-makers to take responsibility in creating conditions needed for continued protection and to fulfil the state’s human rights obligations.

Despite wading uncertainties, we found that unaccompanied girls and young women go to great lengths to adapt and integrate into the host society. Time and stability are needed in distancing oneself from previous traumas, and making space for navigating new norms. With this, new perspectives on SRHR can emerge, and building trustful relationships with healthy boundaries becomes possible. The findings also reflect the essential role and meaning of community and belonging. Several participants found themselves as neither belonging to the country of origin, nor to the host country. In these cases, meaningful relationships and a sense of belonging could be found in the margins. This is reflected in anthropological theories on transitions from adolescence to adulthood, that accent the importance of protections community allow in phases that are otherwise unpredictable [75,76]. Notwithstanding this, as the Council of Europe [37] accented, exclusion and discrimination persist for young refugees, and thus, states must be proactive in promoting their transition into adulthood and inclusion in host societies.

### Strengths and limitations

While this study contributes to an observed gap in the literature, several limitations must be addressed. The analysis relies on retrospective accounts. Memories are known to be reconstructed in relation to the present and future, and both deliberate and inadvertent misrepresentations can be included [77]. Narratives may also reflect participants’ self-image, and how they wish to represent themselves [78]. Yet, although younger individuals might have highlighted different experiences, this retrospective approach allowed for more developed perspectives and language in describing SRHR needs upon arrival.

Further, it is plausible that we failed in reaching the most isolated and at risk members of the included population. Sensitivities related to talking about SRHR also hindered some from taking part. Notwithstanding this, those we did reach showed a generous willingness to participate, and represent a diversity of backgrounds and experiences. Still, they may not be entirely representative. Most demonstrated high levels of agency. They sought independence, striving for financial autonomy and stability. Many also actively worked toward contributing to the wellbeing of others.

Conducting research with marginalised populations requires building trust, while mitigating inevitable power imbalances. Moreover, it has been argued that qualitative researchers in sexuality studies with marginalised populations take on a responsibility that spans beyond the scope of the study [79]. With recruitment and data generation in this study being conducted by MO, who is a doctoral candidate, a registered nurse, and a midwife, this entailed balancing both academic and clinical perspectives throughout. It also required an openness and sensitivity to the needs of participants, offering solicited advice and support where appropriate, as well as assisting them in accessing professional services when needed. All forms of support and assistance were discussed within the research team, with a focus on ethics, and offered before, in conjunction with, and after interviews.

While pre-existing knowledge on the phenomenon of focus may have resulted in a degree of bias within the research team, previously held expertise likely also benefitted the data generation process. The multidisciplinary and interdisciplinary competences within the research team allowed for observing the same phenomena from multiple perspectives, capturing aspects that might otherwise have gone unnoticed. Yet, being positioned as outsiders to the backgrounds and experiences of participants added complexity. Therefore, reflexivity and methodological self-consciousness [66] were guiding. This involved continued methodological and ethical discussions, debriefing of the interviewer, mapping identities of the researchers and participants, consideration of sensitivity, vulnerability, and cultural identity lenses, and addressing reflexive questions [65]. It also involved staying dedicated to flexible interview techniques, allowing for elaboration through participatory engagement while developing the interview-guide. Referral sessions with young adults with lived experience of unaccompanied migration ensured that the guide was grounded in perspectives from the population. This process also minimised the risk of posing questions that could be perceived as sensitive, difficult, excluding, or stigmatising. Furthermore, regular revisiting and refining of interview questions was conducted as long as data generation and analysis remained ongoing.

Finally, while participating in research has been found to be therapeutic for marginalised populations [3,79], talking about traumatic experiences can give rise to unwanted emotions and retraumatisation [80]. Notwithstanding this, several participants shared that taking part in this study and sharing their stories, in some cases for the first time, left them with a sense of relief.

## Conclusion

The present study contributes to the literature by illuminating unaccompanied girls’ shifting SRHR needs, spanning from childhood to adolescence, through the migratory process as unaccompanied girls, and into young adulthood in the host country. Embedded in empirical accounts of their experiences, structural and institutional hindrances and opportunities affecting the fulfilment of their SRHR needs have also been identified.

While many observed needs are universal to all adolescents and young adults, including curiosities regarding identity, sex and intimate relationships, emotions, and bodily functions, we have shown that unaccompanied girls’ SRHR needs are affected on several levels – structural *macro*, interpersonal *meso*, and individual *micro* – and that these needs bleed into adulthood. Within public institutions, an SRHR-informed approach is crucial in eliminating oppressions impeding the fulfilment of SRHR and increasing risks of GBV. Therefore, SRHR literacy and awareness of unaccompanied girls and young women’s specific SRHR needs, as well as tools and skills to address these, are important in assisting professionals who encounter them. This includes competence in identifying risk factors impeding SRHR, and harnessing available opportunities in promoting protective factors.

The study’s findings also indicate the need to evaluate comprehensive sex education and its delivery. To include vulnerable populations, such as unaccompanied girls, adjustments are needed. Where sex education is offered, girls and young women should be facilitated in attending without the presence of the opposite sex. Adapting and communicating information over time is also important, as is covering a broader spectrum of factors that impact SRHR. The failure to provide these adjustments renders the educational space unsafe, leading to attendance avoidance. Furthermore, as participants reported hindrances to healthcare access, more research in this area is needed, from both unaccompanied girls and healthcare professionals’ perspectives.

Finally, our findings highlight the critical need for time and distance, to recover from traumatic experiences, and openly communicate SRHR needs. Thus, public institutions should acknowledge and facilitate this in the recovery process, and ensure access to trauma treatment where needed. Actions must also be taken to provide unaccompanied girls predictability and stability in life, particularly surrounding the asylum process. This is important in remedying SRHR violations and decreasing risks of GBV. Furthermore, public institutions, including social services, healthcare, childcare, and migration authorities, must coordinate remedies to address SRHR needs and increase SRHR literacy among both unaccompanied girls and professionals alike. Failing to do so constitutes a missed opportunity, and risks leaving these girls without guidance in making informed decisions about their bodies and lives. Ultimately, rendering the claiming of their SRHR an individual burden represents a failure on a societal level.

## References

[1] Starrs AM, Ezeh AC, Barker G, Basu A, Bertrand JT, Blum R, et al. Accelerate progress-sexual and reproductive health and rights for all: report of the Guttmacher-Lancet commission. Lancet. 2018;391(10140):2642–92. doi: 10.1016/S0140-6736(18)30293-9 29753597

[2] International Organization for Migration. Glossary on Migration. Geneva: IOM. 2019.

[3] Derluyn I, Lippens V, Verachtert T, Bruggeman W, Broekaert E. Minors travelling alone: a risk group for human trafficking?. Int Migr. 2010;48(4):164–85. doi: 10.1111/j.1468-2435.2009.00548.x 20645474

[4] United Nations Human Rights Office of the High Commissioner. Differentiation between migrants and refugees. https://www.ohchr.org/sites/default/files/Documents/Issues/Migration/GlobalCompactMigration/MigrantsAndRefugees.pdf#:~:text=What%20is%20the%20difference%20between%20a%20‘refugee’%20and%20a%20‘migrant’?. Accessed 2023 October 1.

[5] Kohli RKS, Connolly H. Transitions for young people seeking asylum. Managing Transitions. Bristol University Press. 2009. 73–92. doi: 10.46692/9781847421913.006

[6] UNICEF. Child displacement 2024. https://data.unicef.org/topic/child-migration-and-displacement/displacement/

[7] Asylum applicants considered to be unaccompanied minors by citizenship, age and sex - annual data. Eurostat. 2024. https://ec.europa.eu/eurostat/databrowser/view/MIGR_ASYUNAA/default/table?lang=en

[8] Swedish Migration Agency. Inkomna asylsökande ensamkommande barn flickor 2000-2023. 2024.

[9] United Nations Human Rights Office of the High Commissioner. Your health, your choice, your rights: International and regional obligations on sexual and reproductive health and rights. 2018.

[10] United Nations Committee on Economic, Social, and Cultural Rights. General comment No. 22 (2016) on the right to sexual and reproductive health (article 12 of the International Covenant on Economic, Social and Cultural Rights). United Nations. 2016.

[11] United Nations Committee on the Elimination of Discrimination. General recommendation No. 35 on gender-based violence against women, updating general recommendation No. 19. United Nations. 2017.

[12] Commissioner for Human Rights. Women’s sexual and reproductive health and rights in Europe. Council of Europe. 2017.

[13] United Nations. Transforming our world: The 2030 agenda for sustainable development. United Nations. 2015.

[14] United Nations Population Fund. Sexual and reproductive health and rights: An essential element of universal health coverage. UNFPA. 2019.

[15] Amanu A, Birhanu Z, Godesso A. Sexual and reproductive health literacy among young people in Sub-Saharan Africa: evidence synthesis and implications. Glob Health Action. 2023;16(1):2279841. doi: 10.1080/16549716.2023.2279841 38010100 PMC10795590

[16] Sharma A, Ronan A, Namiba A, Oktariani A, Davies L. Beyond toolkits: sexual and reproductive health and rights literacy requires women-centred structures, services and policies. J Int AIDS Soc. 2022;25(3):e25888. doi: 10.1002/jia2.25888 35257488 PMC8902286

[17] Burke NJ, Joseph G, Pasick RJ, Barker JC. Theorizing social context: rethinking behavioral theory. Health Educ Behav. 2009;36(5 Suppl):55S-70S. doi: 10.1177/1090198109335338 19805791 PMC3548316

[18] Butler J. Bodies that matter on the discursive limits of “sex”. New York: Routledge. 2011.

[19] World Health Organisation. Adolescent and young adult health. Geneva: WHO. 2023.

[20] Thomas S, Thomas S, Nafees B, Bhugra D. ’I was running away from death’- the pre-flight experiences of unaccompanied asylum seeking children in the UK. Child Care Health Dev. 2004;30(2):113–22. doi: 10.1111/j.1365-2214.2003.00404.x 14961864

[21] Tinghög P, Arwidson C, Sigvardsdotter E, Malm A, Saboonchi F. Nyanlända och asylsökande i Sverige: En studie av psykisk ohälsa, trauma och levnadsvillkor. Stockholm: Röda Korsets Högskola. 2016.

[22] Reese Masterson A, Usta J, Gupta J, Ettinger AS. Assessment of reproductive health and violence against women among displaced Syrians in Lebanon. BMC Womens Health. 2014;14(1):25. doi: 10.1186/1472-6874-14-25 24552142 PMC3929551

[23] Svensson P, Carlzén K, Agardh A. Exposure to culturally sensitive sexual health information and impact on health literacy: a qualitative study among newly arrived refugee women in Sweden. Cult Health Sex. 2017;19(7):752–66. doi: 10.1080/13691058.2016.1259503 27894219

[24] Convention on the Rights of the Child. 1989.

[25] United Nations Committee of the Rights of the Child. General comment No. 6. Treatment of unaccompanied and separated children outside their country of origin. United Nations. 2005.

[26] Public Health Agency of Sweden. Hiv-och STI-prevention riktad till migranter. 2014.

[27] Pfarrwaller E, Suris J-C. Determinants of health in recently arrived young migrants and refugees: a review of the literature. Int J Public Health. 2012;9(3). doi: 10.2427/7529

[28] Mason-Jones AJ, Nicholson P. Structural violence and marginalisation. The sexual and reproductive health experiences of separated young people on the move. A rapid review with relevance to the European humanitarian crisis. Public Health. 2018;158:156–62. doi: 10.1016/j.puhe.2018.03.009 29653866

[29] Ivanova O, Rai M, Kemigisha E. A systematic review of sexual and reproductive health knowledge, experiences and access to services among refugee, migrant and displaced girls and young women in Africa. Int J Environ Res Public Health. 2018;15(8):1583. doi: 10.3390/ijerph15081583 30049940 PMC6121882

[30] Ngum Chi Watts MC, Liamputtong P, Carolan M. Contraception knowledge and attitudes: truths and myths among African Australian teenage mothers in Greater Melbourne, Australia. J Clin Nurs. 2014;23(15–16):2131–41. doi: 10.1111/jocn.12335 24028778

[31] Keygnaert I, Guieu A, Ooms G, Vettenburg N, Temmerman M, Roelens K. Sexual and reproductive health of migrants: does the EU care?. Health Policy. 2014;114(2–3):215–25. doi: 10.1016/j.healthpol.2013.10.007 24268324

[32] Borsch AS, de Montgomery CJ, Gauffin K, Eide K, Heikkilä E, Smith Jervelund S. Health, Education and Employment Outcomes in Young Refugees in the Nordic Countries: A Systematic Review. Scand J Public Health. 2019;47(7):735–47. doi: 10.1177/1403494818787099 30067129

[33] Derluyn I, Mels C, Broekaert E. Mental health problems in separated refugee adolescents. J Adolesc Health. 2009;44(3):291–7. doi: 10.1016/j.jadohealth.2008.07.016 19237116

[34] Kohli RKS. Working to Ensure Safety, Belonging and Success for Unaccompanied Asylum‐seeking Children. Child Abuse Review. 2011;20(5):311–23. doi: 10.1002/car.1182

[35] Andersson ES, Øverlien C. Navigating cultural transitions during resettlement: the case of unaccompanied refugee minors. Front Psychol. 2023;14:1080072. doi: 10.3389/fpsyg.2023.1080072 37228345 PMC10203603

[36] Surace GMP. The Future We Want: The Transition to Adulthood of Unaccompanied Minors. Elementa Intersections between Philosophy, Epistemology and Empirical Perspectives. 2022;2(1–2). doi: 10.7358/elementa-2022-0102-sura

[37] Council of Europe. Supporting young refugees in transition to adulthood: Recommendation CMRec(2019)4. 2019.

[38] United Nations Declaration on the Elimination of Violence against Women. 1993.

[39] World Health Organisation. Violence against women: Intimate partner and sexual violence against women. 2011.

[40] United Nations Committee on the Rights of the Child, Committee on the Elimination of Discrimination against Women. Joint general recommendation No. 31 of the Committee on the Elimination of Discrimination against Women/general comment No. 18 of the Committee on the Rights of the Child on harmful practices. 2019.

[41] Sahbani S, Al-Khateeb M, Hikmat R. Early marriage and pregnancy among Syrian adolescent girls in Jordan; do they have a choice?. Pathog Glob Health. 2016;110(6):217–8. doi: 10.1080/20477724.2016.1231834 27680296 PMC5070639

[42] Crenshaw K. Mapping the Margins: Intersectionality, Identity Politics, and Violence against Women of Color. Stanford Law Review. 1991;43(6):1241. doi: 10.2307/1229039

[43] UNFPA. Five reasons migration is a feminist issue. 2018.

[44] Gurnah K, Khoshnood K, Bradley E, Yuan C. Lost in translation: reproductive health care experiences of Somali Bantu women in Hartford, Connecticut. J Midwifery Womens Health. 2011;56(4):340–6. doi: 10.1111/j.1542-2011.2011.00028.x 21733104

[45] Rechel B, Mladovsky P, Ingleby D, Mackenbach JP, McKee M. Migration and health in an increasingly diverse Europe. Lancet. 2013;381(9873):1235–45. doi: 10.1016/S0140-6736(12)62086-8 23541058

[46] Barkensjö [Opperdoes] M, Greenbrook JTV, Rosenlundh J, Ascher H, Elden H. The need for trust and safety inducing encounters: a qualitative exploration of women’s experiences of seeking perinatal care when living as undocumented migrants in Sweden. BMC Pregnancy Childbirth. 2018;18(1):217. doi: 10.1186/s12884-018-1851-9 29879940 PMC5992748

[47] Botfield J, Newman C, Kang M, Zwi A. Talking to migrant and refugee young people about sexual health in general practice. Aust J Gen Pract. 2018;47(8):564–9. doi: 10.31128/AJGP-02-18-4508 30114891

[48] Public Health Agency of Sweden. Migration, sexuell hälsa och hiv-och STI-prevention: En kartläggning av unga migranters sexuella och reproduktiva hälsa och rättigheter i Sverige. 2020.

[49] Åkerman E, Larsson EC, Essén B, Westerling R. A missed opportunity? Lack of knowledge about sexual and reproductive health services among immigrant women in Sweden. Sex Reprod Healthc. 2019;19:64–70. doi: 10.1016/j.srhc.2018.12.005 30928137

[50] Maheen H, Chalmers K, Khaw S, McMichael C. Sexual and reproductive health service utilisation of adolescents and young people from migrant and refugee backgrounds in high-income settings: a qualitative evidence synthesis (QES). Sex Health. 2021;18(4):283–93. doi: 10.1071/SH20112 34412768

[51] Swedish Agency for Health Technology Assessment and Assessment of Social Services. Stöd till ensamkommande barn och unga – effekter, erfarenheter och upplevelser. Systematisk litteraturöversikt och etiska och samhälleliga aspekter. 2018.

[52] National Board of Health and Welfare. Ensamkommande barns och ungas behov - En kartläggning. Stockholm, Sweden: National Board of Health and Welfare. 2013.

[53] Public Health Agency of Sweden. Nationell strategi för sexuell och reproduktiv hälsa och rättigheter (SRHR): En god, jämlik och jämställd sexuell och reproduktiv hälsa i hela befolkningen 2020. 2020.

[54] Public Health Agency of Sweden. Nationell handlingsplan för sexuell och reproduktiv hälsa och rättigheter (SRHR) i Sverige: genomförandet av den nationella SRHR-strategin 2023–2033. 2023.

[55] Public Health Agency of Sweden. Hiv- och STI-prevention och sexuell och reproduktiv hälsa för migranter: En kartläggande litteraturöversikt. 2018.

[56] Government Offices of Sweden. Nationell strategi mot hiv/aids och andra vissa smittsamma sjukdomar. 2017.

[57] Eurostat. People on the move - Statistic on mobility in Europe. 2020.

[58] Lag (2016:752) om tillfälliga begränsningar av möjligheten att få uppehållstillstånd i Sverige. 2016.

[59] Starks H, Trinidad SB. Choose your method: a comparison of phenomenology, discourse analysis, and grounded theory. Qual Health Res. 2007;17(10):1372–80. doi: 10.1177/1049732307307031 18000076

[60] Charmaz K. “With Constructivist Grounded Theory You Can’t Hide”: Social Justice Research and Critical Inquiry in the Public Sphere. Qualitative Inquiry. 2019;26(2):165–76. doi: 10.1177/1077800419879081

[61] Charmaz K. Constructing grounded theory. 2 ed. Thousand Oaks, California: SAGE Publications. 2014.

[62] Brinkmann S, Kvale S. InterViews: Learning the craft of qualitative research interviewing. 3 ed. Los Angeles: SAGE Publications. 2015.

[63] Saldaña J. The coding manual for qualitative researchers. 4 ed. Los Angeles: SAGE Publications. 2021.

[64] Birks M, Mills J. Rendering analysis through storyline. In: Charmaz K, Bryant A, editors. The SAGE handbook of current developments in grounded theory. London: SAGE Publications. 2019. p. 243–58.

[65] Joseph FI, Earland J, Ahmed MA. Experience of conducting sensitive qualitative research as a cultural outsider: formulation of a guide for reflexivity. Int J Qualitative Methods. 2021;20. doi: 10.1177/16094069211058616

[66] Charmaz K. The Power of Constructivist Grounded Theory for Critical Inquiry. Qualitative Inquiry. 2016;23(1):34–45. doi: 10.1177/1077800416657105

[67] World Medical Association. Declaration of Helsinki. 2018.

[68] Kågesten A, Gibbs S, Blum RW, Moreau C, Chandra-Mouli V, Herbert A, et al. Understanding factors that shape gender attitudes in early adolescence globally: a mixed-methods systematic review. PLoS One. 2016;11(6):e0157805. doi: 10.1371/journal.pone.0157805 27341206 PMC4920358

[69] Grotti V, Malakasis C, Quagliariello C, Sahraoui N. Shifting vulnerabilities: gender and reproductive care on the migrant trail to Europe. Comp Migr Stud. 2018;6(1):23. doi: 10.1186/s40878-018-0089-z 30101080 PMC6061107

[70] Blum RW, Astone NM, Decker MR, Mouli VC. A conceptual framework for early adolescence: a platform for research. Int J Adolesc Med Health. 2014;26(3):321–31. doi: 10.1515/ijamh-2013-0327 24486726 PMC4476282

[71] Freedman J, Sahraoui N, Tastsoglou E. Gender-based violence in migration: interdisciplinary, feminist and intersectional approaches. Cham, Switzerland: Palgrave Macmillan. 2022.

[72] Collins CH, Zimmerman C, Howard LM. Refugee, asylum seeker, immigrant women and postnatal depression: rates and risk factors. Arch Womens Ment Health. 2011;14(1):3–11. doi: 10.1007/s00737-010-0198-7 21153849

[73] O’Connor MK, Wood E, Frazier T. Reducing risk and building agency for vulnerable asylum seekers. J Emerg Manag. 2023;20(8):91–102. doi: 10.5055/jem.0681 36825634

[74] Montgomery E. Trauma, exile and mental health in young refugees. Acta Psychiatr Scand Suppl. 2011;(440):1–46. doi: 10.1111/j.1600-0447.2011.01740.x 21824118

[75] Turner E. Communitas: The anthropology of collective joy. New York, New York: Palgrave Macmillan. 2012.

[76] van Gennep A. Les rites de passage [The rites of passage]. Paris, La Haye: Mouton. 1969.

[77] Gemignani M. Memory, remembering, and oblivion in active narrative interviewing. Qualitative Inquiry. 2014;20(2):127–35. doi: 10.1177/1077800413510271

[78] Pearlman W. Memory as a Field Site: Interviewing Displaced Persons. Int J Middle East Stud. 2017;49(3):501–5. doi: 10.1017/s0020743817000356

[79] Bahner J, Lindroth M. Researchers with benefits? methodological and ethical challenges and possibilities in sexuality research within marginalised populations. Int J Qualitative Methods. 2023;22. doi: 10.1177/16094069231171095

[80] Pardee L, Hung Y, Rahman L, Patel G, Munoz N, Ades V. A trauma-informed care approach to conducting forensic medical and psychological evaluations for female asylum seekers. Int J Migration Health Social Care. 2025;21(2):324–42. doi: 10.1108/ijmhsc-05-2024-0052

